# Identification of mutations in the ATP7B gene in 14 Wilson disease children: Case series

**DOI:** 10.1097/MD.0000000000025463

**Published:** 2021-04-23

**Authors:** Jiuxiang Wang, Lulu Tang, Anqi Xu, Shijie Zhang, Hailin Jiang, Pei Pei, Hongmei Li, Tingting Lv, Yue Yang, Nannan Qian, Keegan Naidu, Wenming Yang

**Affiliations:** a Experimental Center of Clinical Research; b Department of Neurology; c Nangjing Red Cross Blood Center, Nangjing; d Clinical Laboratory Center, The First Affiliated Hospital of Anhui University of Chinese Medicine, Hefei; e Department of Rehabilitation Medicine, Laian People's Hospital, Chuzhou; f School of International Studies, Anhui Medical University, Hefei, Anhui, PR China.

**Keywords:** ATPase copper transporting beta, bioinformatics, mutation, single nucleotide polymorphism, Wilson disease

## Abstract

**Introduction:**

Wilson Disease (WD) is an autosomal recessive inherited metabolic disease caused by mutations in the ATPase copper transporting beta gene (*ATP7B*). WD can cause fatal neurological and hepatic disorders if not diagnosed and treated.

**Objective:**

To analyze the disease-causing mutations of 14 Chinese WD children, 11 of whom are diagnosed with hepatic disorders, 2 with neurological degeneration and 1 with both hepatic and neurological disorders.

**Methods:**

All *ATP7B* coding regions were analyzed by Sanger sequencing. Single nucleotide polymorphisms (SNPs) functional impacts were assessed by combining the results of four bioinformatics tools (Poly-phen-2, SIFT, PANTHER-PSEP and PhD-SNPs) in an index that reflects the combined probability (cP_del_) of an amino acid change to be deleterious to the protein function.

**Results:**

Two novel variants involved in WD development, c.1448_1455del (p.Arg483SerfsX19) and c.4144G>T (p.Glu1382Stop), and 11 previously reported mutations were detected. Both new variants result in shortened and dysfunctional ATP7B proteins. cP_del_ score suggests that SNPs may be deleterious to the ATP7B functionality.

**Conclusions:**

This study enriches the library of the *ATP7B* mutations that lead to WD and can be used as a basis for genetic counseling, for WD prevention and clinical and prenatal diagnosis. Those SNPs that are believed to be harmless to ATP7B protein may be involved in the pathogenesis of WD.

## 1 Introduction

Wilson's disease (WD; MIM 277900) is an autosomal recessive copper metabolism disorder, including inadequate incorporation of copper into apoceruloplasmin and impaired biliary copper excretion. This metabolic disorder leads to cooper accumulation mainly in the liver and brain and concomitantly, which causes hepatic, neurological or psychiatric impairment, Kayser–Fleischer (KF) rings and other complex clinical manifestations. Although copper metabolism disorder begins at birth, symptoms do not usually develop until the age of 3. In most patients, symptoms appear between 5 and 35 years of age.^[1]^ Pediatric WD patients mainly show liver symptoms, whereas neuropsychiatric symptoms predominate in adults.^[2]^

ATPase copper transporting beta *(ATP7B)* is the know WD causative gene, located on chromosome 13q14.3 and encodes a copper-transporting P-type ATPase that contains 1465 residues. This protein is formed by 6 mental binding units, 8 transmembrane domains (TM), an actuator domain (A-domain), a phosphorylation domain (P-domain) and a nucleotide binding domain (N-domain).^[3]^ More than 700 mutations and about 800 single-nucleotide polymorphisms (SNPs) have been identified in *ATP7B*, but only a few have been studied experimentally.^[4]^ The study of function, stability and traffic of ATP7B is difficult for it is a large transmembrane protein. Combined probability (cP_del_) can be a useful algorithm to estimate an amino acidic change in proteins. This algorithm is able to combine the results of the four most used bioinformatics tools (Poly-phen-2, SIFT, PANTHER, and PhD-SNPs) to evaluate the SNPs effect on the protein function.^[5–8]^ cP_del_ values of the WD-causing mutations with deleterious effects experimentally verified are higher (0.98) than the non-deleterious mutations (0.88) and non-disease-causing variants (NDVs) (0.21).^[3]^

The prevalence of WD is estimated at 1 in 30,000, and the heterozygous carrier rate is close to 1 in 90 in many populations.^[9,10]^ Early diagnosis and treatment are critical for preventing disease progression and irreversible sequelae.^[11]^ Genetic testing of *ATP7B* is a reliable tool for diagnosing WD, especially for early onset. In this study, we reported two new mutations, c.1448_1455del (p.Arg483Serfs X19) and c.4144G>T (p.Glu1382 Stop), as well as other 11 previously reported mutations were identified by direct DNA sequencing analysis of the entire coding regions of *ATP7B* in 14 WD cases. One SNP obtained moderate cP_del_ scores, suggesting that SNPs previously considered non-pathogenic may impair ATP7B function.

## 2 Materials and methods

### 2.1 Patients

Fourteen cases of WD children were identified at the First Hospital of Anhui University of Chinese Medicine. This study followed the tenets of the Declaration of Helsinki and was approved by the ethics committee of The First Affiliated Hospital of Anhui University of Chinese Medicine. Written consent was obtained from patients or their guardians before participating in the genetic investigation and after explanation of the nature and possible consequences of this study.

### 2.2 Mutation detection

Venous blood samples were collected from patients and genomic DNA was isolated according to the method described by Ren et al.^[12]^ The coding exons of ATP7B from exon 1 to 21 and the exon-intron boundaries were amplified using DNA from patients with the corresponding primers (Supplemental Table 1). PCR products were gel-purified (Watson Biotechnologies, Inc., Shanghai,China) and sequenced by BigDye 3.1 (Applied Biosystems, Foster City, CA). Raw data were direct sequencing analysis with the ABI PRISM 3100 Genetic Analyzer and Sequencing Analysis 5.3.1 software. The sequencing results were compared to the reported cDNA reference sequence (NM_000053).

### 2.3 SNPs functional prediction analysis

The combined probability (cP_del_) of an amino acid changes deleterious to protein function was predicted as Polimanti et al described with slight modification.^[3]^ We obtained information from four software programs to calculate cP_del_: Polyphen-2,^[5]^ SIFT,^[6]^ PhD-SNP^[8]^ PANTHER-PSEP.^[7]^ PANTHER-PSEP estimates the likelihood of an amino acid change to impact the structure or function of a protein by calculating the time period preserved in the evolutionary lineage leading to the protein. We assigned a score of 0 for a likely benign change (time < 200 million years, my), a score of 0.5 for a possibly damaging change (450my > time > 200my) and a score of 1 for a probably damaging change (time > 450my). Hence, we calculate cP_del_ according to following equation:

cP_del_ = mean [Score Polyphen 2; 1-Score_SIFT_; P_PANTHER-PSEP_ (probably damaging = 1, possibly damaging = 0.5, probably benign = 0); Prediction_PhD-SNP_ (Neutral = 0; Disease = 1)].

The cP_del_ score ranges from 0 (with no impact on function) to 1 (complete loss of function).

## 3 Results

### 3.1 Case reports

Fourteen children underwent a careful physical examination and were diagnosed with WD (Table 1). Eleven cases presented mainly liver dysfunction and were diagnosed before 10 years of age, while cases 12 and 13 presented neurological symptoms and case 14 showed both hepatic and neurological disorders.

**Table 1:** Clinical data of pediatric patients with WD at diagnosis.

Patients No	Sex	Age at diagnosis	ALT (U/L)	AST (U/L)	Ceruloplasmin (mg/dl)	K-F ring	Symptoms
1	Male	7	ND	ND	ND	−	No apparent symptoms
2	Female	4	248.3	168.3	0.64	−	Abdominal pain
3	Male	3	61	42	0.096	ND	No apparent symptoms
4	Male	8	30.4	62.4	1	+	No apparent symptoms
5	Male	4	175	131	ND	−	ND
6	Female	8	ND	ND	ND	ND	No apparent symptoms
7	Male	7	165	72	ND	−	ND
8	Female	6	109	143	ND	−	No apparent symptoms
9	Female	4	105	72	0.03	−	No apparent symptoms
10	Male	6	149	86	ND	ND	ND
11	Female	6	81	62	ND	ND	ND
12	Female	11	16	22	ND	+	A
13	Male	12	63	54	ND	+	cough and fever
14	Male	12	194	73.8	0.03	ND	B

Cases 1, 3, 4, 8, and 9 were discovered after physical examination of school children without obviously symptoms. Case 12 presented dribbling, involuntary tremor and KF rings, and magnetic resonance imaging showed a low symmetrical signal from the bilateral basal ganglia and the substantia nigra. Case 13 showed map-like brain waves, KF rings, low ceruloplasmin when he had fever and cough at age of 12. His parents reported he experienced Henoch-Schonlein purpura. Case 14 visited our hospital due to a sudden loss of consciousness, limbs convulsion and involuntary staring when he was 12 years old. Physical examination revealed KF rings, abnormal intermittent epileptic discharge and deffective electroencephalogram. His parents reported that the development of his intelligence and language functions was retarded at 2 years of age.

### 3.2 Analysis of mutations in the *ATP7B* gene

**Figure 1:**
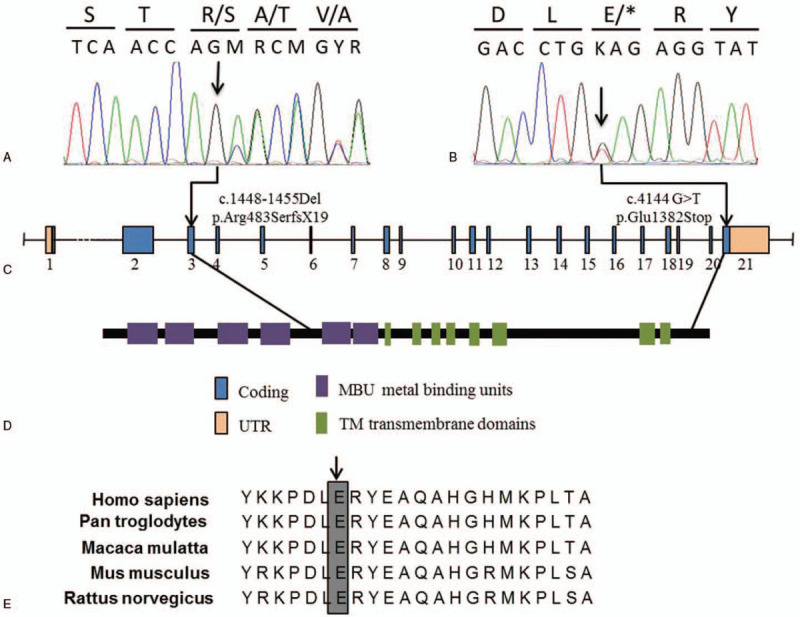
Mutation analysis of *ATP7B* gene. (A.B) Two mutations of ATP7B gene, c.1448–1455Del and c.4144 G>T in two WD patients respectively, were shown on the sequencing chromatograph. (C) Genomic structure of the human ATP7B gene. (D) Protein structure of the ATP7B protein. (E) Multiple amino acid sequences alignment of the ATP7B protein were analyzed with Clustalx software (Ver. 1.83). The amino acid sequences of *Homo sapiens* and four other species were obtained from the NCBI public database (http://www.ncbi.nlm.nih.gov/protein).

Thirteen mutations were detected in our present analysis (Table 2), including two unprecedented mutations (c.1448_1455del and c.4144G>T) (Fig. 1) and eleven previously reported. Of those, eleven were found to be compound heterozygotes, and c.2333G>T found to be homozygous (Table 2). The c.2333 G>T (p.Arg778Gln) variant is a hotspot mutation in Chinese people and was identified in patients 1, 3, 4, 11 and 12 (Table 2). In contrast to other patients, only one missense mutation (c.2621C>T) in *ATP7B* was identified in case 14. The other variants (c.2495A>G, c.2855G>T, c.3419C>T, c.3903+6 C>T) identified in this case were previously reported as NDVs (Table 3).

**Table 2:** Distribution of mutations detected in the *ATP7B* gene.

Patients no	Mutation	Amino Acid	Area of Protein	Exon	Cellular Localization
1	c.2333G>T	p.Arg778Gln	TM4	8	Transmembrane
	c.3809A>G	p.Asn1270Ser	ATP hinge	18	cytoplasm
2	c.2975C>T	p.Pro992Leu	TM6	13	Transmembrane
	c.2668G>A	p.Val890Met	TM5	11	cytoplasm
3	c.2333G>T	p.Arg778Gln	TM4	8	Transmembrane
	c.2621C>T	p.Ala874Val	A-domain	11	cytoplasm
4	c.2333G>T	p.Arg778Gln	TM4	8	Transmembrane
	c.2333G>T	p.Arg778Gln	TM4	8	transmembrane
5	c.2621C>T	p.Ala874Val	A-domain	11	Cytoplasm
	c.2621C>T	p.Ala874Val	A-domain	11	cytoplasm
6	c.2304dupC	p.Met769HisfsX26	TM4	8	Transmembrane
	c.2975C>T	p.Pro992Leu	TM6	13	transmembrane
7	c.1448_1455del^∗^	p.Arg483Serfs X19	Mbu4/Mbu5	3	Cytoplasm
	c.2621C>T	p.Ala874Val	A-domain	11	cytoplasm
8	c.2975C>T	p.Pro992Leu	TM6	13	Transmembrane
	c.4144G>T^∗^	p.Glu1382Stop	After TM8	21	cytoplasm
9	c.3517G>A	p.Glu1173Lys	ATP bind	16	Cytoplasm
	c.3955C>T	p.Arg1319Stop	TM7	19	transmembrane
10	c.2294A>G	p.Asp765Gly	TM4	4	Transmembrane
	c.2752G>A	p.Asp918Asn	TM5	12	transmembrane
11	c.2333G>T	p.Arg778Gln	TM4	8	Transmembrane
	c.2755C>G	p.Arg919Gly	TM5	12	Transmembrane
12	c.2333G>T	p.Arg778Gln	TM4	8	Transmembrane
	c.2975C>T	p.Pro992Leu	TM6	13	Transmembrane
13	c.2662A>C	p.Thr888Pro	TM4/A-domain/TM5	11	Cytoplasm
	c.3316G>A	p.Val1106Ile	ATP loop	15	Cytoplasm

**Table 3:** Variations found in case 14 WD Patient.

Exon	Variant name	Amino acid change	Genotype	Area of protein	Cellular localization
10	c.2495A>G	p.Lys832Arg	Heterozygotes	TM4/A-domain/TM5	Cytoplasm
11	c.2621C>T	p.Ala874Val	Heterozygotes	TM4/A-domain/TM5	Cytoplasm
12	c.2855G>T	p.Arg952Lys	Homozygotes	TM5/TM6	Lumen
16	c.3419C>T	p.Val1140Ala	Heterozygotes	N-domain: ATP bind	Cytoplasm
18	c.3903+6 C>T	Splice	Heterozygotes	Bet ATP hinge/TM7	Transmembrane

ATP7B is a transmembrane enzyme that contains 1465 amino acids. The new *ATP7B* deletion variant c.1448_1455del was identified in case 7, which had liver disorders. This mutation changes the reading frame and introduces an early termination codon in the N-terminal region (Fig. 1 A and C). Consequently, variant c.1448_1455del results in a shortened and functionless protein that lacks 2 mental binding units domains, 8 TM domains and several essential dysfunctional regions (Fig. 1D). The other new variant *ATP7B* c.4144G>T, was identified in case 8. This mutation introduces a termination codon at position 1382, resulting in a truncated polypeptide of 1381residues (Fig. 1 C and D). Naturally, position 1382 is occupied by a Glu residue that is conserved in 5 vertebrate species, including *Pan troglodytes*, *Macaca mulatta*, *Mus musculus* and *Rattus norvegicus* (Fig. 1E).

### 3.3 cP_del_ revealed that some SNPs may be deleterious to the ATP7B function

As expected, WD-causing variants obtained high cP_del_ values (0.746; Table 4), which predict undesirable effects on the ATP7B function. This result indicates that cP_del_ is a reliable bioinformatics predictor of the impact of SNPs on the ATP7B function. The previously reported c.2621C>T (p.Ala874Val) mutation and 4 NDVs were detected in case 14. cP_del_ score of c.2621C>T (p.Ala874Val) variant is 0.999. Considering that SNPs contribute to the pathogenesis of WD, we estimated the cP_del_ of the three SNPs using data obtained by Polyphen-2, SIFT, PANTHER-PSEP and PhD-SNP softwares (Table 4). The score of variant c.2495A>G (p.Lys832Arg) was 0.423 in Polyphen-2 and 0.087 in SIFT1. PANTHER-PSEP estimated that Lys832 has been preserved for 750 million years and that Lys832Arg could probably be a damaging mutation that obtained a score of 1.000. PhD-SNP predicted that Lys832Arg is a neutral causative variant that obtained 0 score. c.2495A>G (p.Lys832Arg) variant obtained a cP_del_ value of 0.584. The SNP c.2855G>T (p.Arg952Lys) obtained 0.000 and 1.000 scores in Polyphen-2 and SIFT softwares, respectively. According to the PhD-SNP software, this variant would be a disease causing SNP, but probably a benign change (6 million years) according to PANTHER-PSEP. At last, the cP_del_ value of variant c.2855G>T (p.Arg952Lys) is 0.25 (Table 4). The SNP c.3419C>T (p.Val1140Ala) obtained 0.000, 0.914, neutral and probably benign (6 million years) as the results of the 4 predictors, resulting in a cP_del_ score of 0.022 (Table 4). These results showed that each SNP has different effects on protein function and that SNP with moderate cP_del_ scores can be deleterious to ATP7B function.

**Table 4:** cP_del_ outcomes of *ATP7B* variants.

Varients	Disease Status	Poly phen-2	SIFT	PhD-SNP	Panther-PSEP	cP_del_	Experimental deleterious^18^
Asp765Gly	DV	1.000	0.001	Disease	Probably damage	1.000	Low transport, reduced expression
Arg778Gln	DV	1.000	0.000	Disease	Probably damage	1.000	Low stability, abnormal localization
Lys832Arg	NDV	0.423	0.087	Neutral	Probably damage	0.584	
Ala874Val	DV	1.000	0.004	Disease	Probably damage	0.999	Misfolded, mistarget, no activity
Thr888Pro	DV	0.998	0.001	Disease	Probably damage	1.000	
Val890Met	DV	1.000	0.003	Disease	Probably damage	0.999	
Asp918Asn	DV	1.000	0.000	Disease	Probably damage	1.000	
Arg952Lys	NDV	0.000	1.000	Disease	Probably benign	0.25	
Pro992Leu	DV	1.000	0.001	Disease	Probably damage	1.000	Inactivation in yeast assay, affected foldings, very low transport
Val1106Ile	DV	0.863	0.156	Disease	Possibly damage	0.802	Inactivation in yeast assay
Val1140Ala	NDV	0.000	0.914	Neutral	Probably benign	0.022	Full yeast complementation
Glu1173Lys	DV	0.983	0.002	Neutral	Probably damage	0.746	
Asn1270Ser	DV	1.000	0.000	Disease	Probably damage	1.000	Inactivation in yeast assay, low transport

## 4 Discussion and conclusion

WD may present at any age with variable symptoms of liver disease. Copper accumulates in liver tissue during childhood, so that abnormal liver function test results may occur long before symptom onset. 12 WD individuals were showed elevated serum aminotransferases concentrations due to healthy test or WD dis-associated lab examinations. Diagnostic testing for should be taken once increased aminotransferases are observed in WD pediatric cohor.

In this study, we analyzed *ATP7B* mutations in 14 WD children and identified two, c.1448_1455del (p.Arg483SerfsX19) and c.4144G>T (p.Glu1382stop), which had not been previously reported. The mutation c.1448_1455del was found in case 7 and occurred as a compound heterozygote with mutation c.2621C>T. The other new mutation was found in case 8 as a compound heterozygote with c.2975C>T (p.Pro992Leu). c.2621C>T is one of the most frequent mutations in Chinese people and was found in cases 3, 5 and 14. Case 5 is a c.2621C>T homozygote with hepatic symptoms, case 3 showed hepatic symptons, while case 14 showed severe neurological degeneration than liver disorders. In case 14, only c.2621C>T mutation was identified, indicating that other mutations may have occurred in *ATP7B* or other genes. Nevertheless, these results suggested that the phenotype of the c.2621C>T mutation presented a hepatic WD with a relatively early onset and that the WD symptoms are a consequence of each mutation-specific phenotype.

SNPs are the largest source of variation in the human genome. Approximately 800 SNPs have been identified in *ATP7B* gene, some of which can modulate the cellular and biochemical properties of the ATP7B protein.^[4,13]^ cP_del_ is an index that combines the outcomes of four popular bioinformatics tools (Polyphen-2, SIFT, PhD-SNP and PANTHER-PSEP) widely used to predict the functional impact of SNPs on gene function.^[3]^ In case 14, 1 mutation c.2621C>T (p.Ala874Val) and 3 SNPs were identified in *ATP7B* gene; however, there may be other mutations, such as splicing mutation that impair protein function. We observed that cP_del_ scores varied between SNPs. Val1140Ala is an experimentally validated mutation that is deleterious to the ATP7B function. This mutation obtained a cP_del_ score of 0.022. The variant Lys832Arg obtained a cP_del_ score of 0.548, which is more than twice as high as that obtained by the Arg952Lys variant (0.25). The Lys832 residue is located in the actuator domain between TM segments 4 and 5, constitutes the antiparallel β3–strand and affects the conformational dynamics of the A-domain.^[14,15]^ Moreover, R832 has been identified as a loss-of-function SNP in *Drosophila melanogaster* ATP7.^[15,16]^ These findings suggest that SNPs may contribute to WD progressing, and that cP_del_ score could be an easy and useful tool for quickly assessing the effect of SNPs on the ATP7B function.

In summary, genetic analysis can be a very useful tool for diagnosis WD, as biochemical tests may be insufficiently sensitive in very young children. Our study enriched the library of *ATP7B* mutations involved in WD development. ATP7B is a large copper-transporting ATPase that plays a key role in regulating copper homeostasis. Given that it is difficult to trace new genetic variants in *ATP7B* experimentally, the cP_del_ bioinformatic method can be a useful and simple tool for the first screening of mutations in the *ATP7B* gene and to valuate the SNPs effect on ATP7B function.

## Acknowledgment

We are grateful to all the participants and their guardians in the study.

## Author contributions

**Data curation:** Jiuxiang Wang, Hailin Jiang, Hongmei Li, Tingting Lv, Nannan Qian.

**Formal analysis:** Lulu Tang, Anqi Xu, Shijie Zhang, Hailin Jiang, Hongmei Li, Nannan Qian.

**Funding acquisition:** Pei Pei, wenming yang.

**Investigation:** Lulu Tang, Yue Yang, Keegan Naidu.

**Methodology:** Anqi Xu.

**Project administration:** wenming yang.

**Resources:** Pei Pei, wenming yang.

**Software:** Lulu Tang, Shijie Zhang, Pei Pei, Tingting Lv.

**Validation:** Shijie Zhang.

**Visualization:** Anqi Xu.

**Writing – original draft:** Jiuxiang Wang.

**Writing – review & editing:** Jiuxiang Wang, Lulu Tang.

## Supplementary Material

Supplemental Digital Content
