# Supplementary material for: Identification of mutations in the ATP7B gene in 14 Wilson disease children: Case series
Source: Medicine (Baltimore). 2021 Apr 23;100(16):e25463. doi: 10.1097/MD.0000000000025463 (PMC8078297; doi:10.1097/MD.0000000000025463)
Supplement: Supplemental Digital Content [file medi-100-e25463-s001.docx]

**Supplemental Table 1. Primers for *ATP7B* mutation screening**

| Exons | Name | Sequences(5’-3’) | Size |
| --- | --- | --- | --- |
| 1 | 1F  1R | TCCTCTCCCGGGACTTTAAC  GCGAGTAAGCGCCGAAC | 231 |
| 2 | 2-1F  2-1R | CCAGAGAAGCTGGGATGTTG TGACCACATGGCTTCCTTG | 792 |
|  | 2-2F  2-2R | TGACATGGGATTTGAAGCTG GCAGGGCTCACCTATACCAC | 770 |
| 3 | 3F  3R | CTCACCAAGAGCCCTGAAAC TTGCTGGGTATTCTGAAGGG | 420 |
| 4 | 4F  4R | TGGGAAGATGTGTTTCTTTGTTC CAGACACGTCCAAGATGGG | 363 |
| 5 | 5F  5R | CTCCCTGGACTGGCTTTCAC TTCCATGGGAAAAGTTGAAG | 362 |
| 6 | 6F  6R | CACAAAGTCTACTGAGGCACTTTTAG CAGAGTTGGGCCCAGGTAG | 275 |
| 7 | 7F  7R | CTGTGTCCTCAGAAGGGGAG TTAGCGGGCAGAATATCTGAG | 329 |
| 8 | 8F  8R | ATAAACGCCCATCACAGAGG GCACCTTAATTATATGGAGGTTTCC | 524 |
| 9 | 9F  9R | ACCACGCTTGTGACTCTCAG TCAATACAACATGGGCATCTG | 253 |
| 10-11 | 10-11F  10-11R | AACAGCTGGCCTAGAACCTG TCTGATTTCCCAGAACTCTTCAC | 540 |
| 12 | 12F  12R | CAATCTTTATCCATGCTTGTGG TGAATAATTAAAGCCCAGTGAATC | 317 |
| 13 | 13F  13R | TCCTTATGTGATTAGAGTTCTGGG GAGTGGCTCTCAGGCTTTTC | 360 |
| 14 | 14F  14R | GTTGGGTGAAGTTCTGCCTC TGGTTTTCCAGACCACACAG | 383 |
| 15 | 15F  15R | CTTCACCCTGTGTCCCTGTC CAGAGGCAATCACTGCTGG | 349 |
| 16 | 16F  16R | GCTGTTAAAAGGATTGCATGG AAAGGAGGACTCTTTTGCCTG | 302 |
| 17 | 17F  17R | TTGCAAGTGTGGTATCTTGG TGGAGAGAAAAGCATCCAGC | 343 |
| 18-19 | 18-19F  18-19R | CAACATCACTGACTGGACCC CCACTCACTAACCCCAGCAG | 602 |
| 20 | 20F  20R | GTGCCTGAAGCCCTCTCC TTGTCCCAGGTGAATGAATG | 283 |
| 21 | 21F  21R | AGGCCTTCACCAGGCTTAG TATCCAGGGAGCGGAAGTC | 474 |
